# Transcriptional Reprogramming of CD11b^+^Esam^hi^ Dendritic Cell Identity and Function by Loss of Runx3

**DOI:** 10.1371/journal.pone.0077490

**Published:** 2013-10-15

**Authors:** Joseph Dicken, Alexander Mildner, Dena Leshkowitz, Ivo P. Touw, Shay Hantisteanu, Steffen Jung, Yoram Groner

**Affiliations:** 1 Department of Molecular Genetics, The Weizmann Institute of Science, Rehovot, Israel; 2 Department of Immunology, The Weizmann Institute of Science, Rehovot, Israel; 3 Bioinformatics Unit of The Israel National Center for Personalized Medicine, The Weizmann Institute of Science, Rehovot, Israel; 4 Department of Hematology, Erasmus Medical Center, Rotterdam, The Netherlands; New York University, United States of America

## Abstract

Classical dendritic cells (cDC) are specialized antigen-presenting cells mediating immunity and tolerance. cDC cell-lineage decisions are largely controlled by transcriptional factor regulatory cascades. Using an *in vivo* cell-specific targeting of Runx3 at various stages of DC lineage development we show that Runx3 is required for cell-identity, homeostasis and function of splenic Esam^hi^ DC. Ablation of Runx3 in DC progenitors led to a substantial decrease in splenic CD4^+^/CD11b^+^ DC. Combined chromatin immunoprecipitation sequencing and gene expression analysis of purified DC-subsets revealed that Runx3 is a key gene expression regulator that facilitates specification and homeostasis of CD11b^+^Esam^hi^ DC. Mechanistically, loss of Runx3 alters Esam^hi^ DC gene expression to a signature characteristic of WT Esam^low^ DC. This transcriptional reprogramming caused a cellular change that diminished phagocytosis and hampered Runx3^-/-^ Esam^hi^ DC capacity to prime CD4^+^ T cells, attesting to the significant role of Runx3 in specifying Esam^hi^ DC identity and function.

## Introduction

 Classical dendritic cells (cDC) are a sparsely distributed, migratory group of bone-marrow- (BM) derived immune cells specialized in antigen uptake and presentation [1,2] and as such important mediators of immunity and tolerance [3,4]. cDC are divided into two main groups: migratory DC (mDC) and stationary DC (sDC). mDC are scattered in most body organs including lung, gut and skin and act as sentinels by sampling environmental pathogens. Upon activation, mDC migrate to secondary lymphoid organs and present processed antigens to T cells. sDC, on the other hand, sample and process antigens directly in the lymphoid organs. These sDC are heterogeneous with respect to their cell-surface markers and function and have based on surface molecules been divided into three major types: XCR1^+^ CD8α^+^/CD4^-^/CD11b^-^, CD4^+^/CD11b^+^/CD8α ^-^ and CD4^−^/CD8α^−^ CD11b^+^ [5]. In addition, splenic CD8α ^+^ and CD4^+^ subsets differ in MHCI and MHCII processing pathways, reflected in their ability to preferentially induce CD4 or CD8 T cell responses [6]. 

Despite their phenotypic and functional heterogeneity all splenic cDC are derived from a common DC precursor (CDP) [7]. BM CDP give rise to plasmacytoid DC (pDC) and circulating pre-DC that seed the peripheral tissues [7]. The current classification defines two distinct subclasses of splenic CD11b^+^ cDC, as CD11b^+^Esam^hi^ and CD11b^+^Esam^low^ cells [8]. Gene expression profiling and functional analysis of these two CD11b^+^ cDC subclasses have suggested that CD11b^+^Esam^low^ DC are related to monocytes rather than to cDC and therefore could have derived from the monocyte/dendritic cell precursor (MDP) without involving a CDP intermediate [8]. Generation of CD11b^+^Esam^hi^/CD11b^+^Esam^low^ DC is controlled by the Notch2 pathway, so that loss of Notch2 signaling selectively affects the development of CD11b^+^Esam^hi^ DC [8].

Several transcription factors (TF) affect DC lineage development and function. Mice lacking the TF Id2, Irf8 or Batf3 are deficient in CD8^+^ DC [9-11] and loss of Irf4 TF affects CD4^+^ DC development [12]. On the other hand, loss of the cDC-specific TF zDC (Zbtb46) [13,14] did not impair DC development, but was associated with increased activation of naive cDC [15]. 

Runx3 TF is highly expressed in mature BM derived cultured DC and mediates their response to TGF-ß [16]. Here we explored Runx3 function throughout DC lineage development using mice that lack Runx3 specifically in DC and their progenitors. Runx3 ablation at defined developmental DC stages led to largely impaired splenic CD4^+^/CD11b^+^ DC compartment. Combined chromatin immunoprecipitation sequencing (ChIP-seq) and gene expression analysis revealed that Runx3 acts as a key gene expression regulator of CD11b^+^Esam^hi^ DC homeostasis. Accordingly, loss of Runx3 altered gene expression profile of the residual Runx3 deleted (Runx3^Δ^) Esam^hi^ DC to a profile characteristic of WT Esam^low^ DC subtype. Moreover, this transcriptional reprogramming yielded functionally impaired Runx3^Δ^ Esam^hi^ DC compromising their ability to prime CD4^+^ T cells. The results defined Runx3-regulated target genes that participate in Runx3-mediated DC lineage development and function. 

## Methods

### Mice

**Figure 1:**
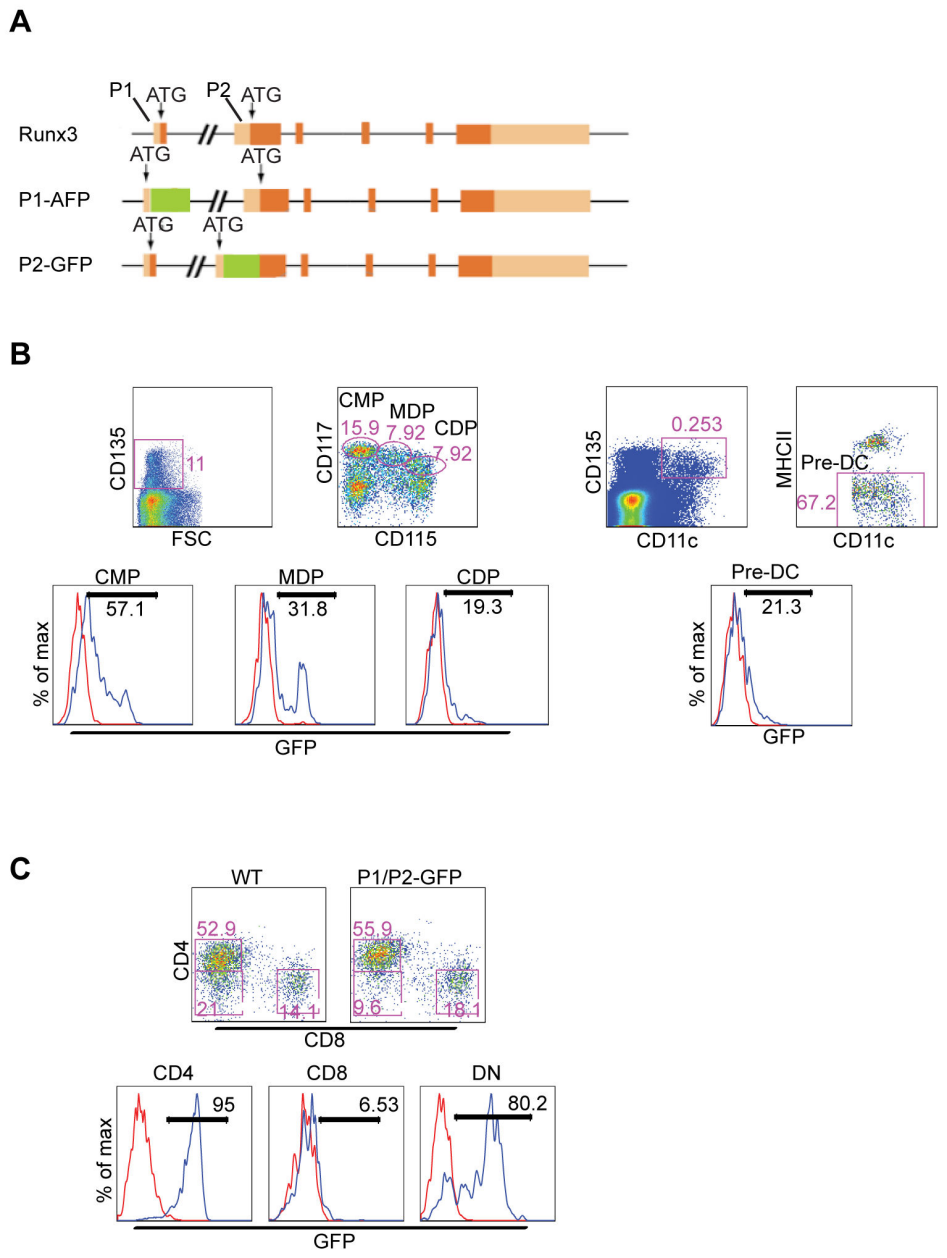
Runx3 expression is restricted to splenic CD4^+^/CD11b^+^ DCs and to fraction of CMP and MDP. (A) Schematic representation of the Runx3 gene and the targeted alleles used to generate the Runx3-GFP reporter mice. P1 and P2 represents promoter 1 and 2 respectively. (B) Flow cytometry analysis of WT or Runx3P1/P2-GFP BM cells gated for lineage negative cells and stained for myeloid progenitor markers to identify GFP expression in CMP, MDP, CDP and preDC. WT littermate and Runx3P1/P2-GFP mice are marked by red and blue lines, respectively. (C) Flow cytometry analysis of cells isolated from WT or Runx3P1/P2-GFP mice. Dot plot of isolated splenic CD11c+MHCII+ DC stained for CD4+ and CD8+ DC subsets (upper panel). CD4+ and DN DC express Runx3 (lower panel histograms). CD8+ DC are GFP negative indicating lack of Runx3 expression. All data in this figure are representative of three independent experiments with one to two mice per group.

 The Runx3^P1/P2-GFP^ KI and Runx3^-/-^ (KO) mice were previously described [17] [18] (Figure 1A). Mice lacking Runx3 in specific DC progenitors were produced by crossing Runx3^fl/fl^ mice [17] onto Cebpα-Cre mice [19] or CD11c-Cre mice [20]. This mating scheme generated Runx3^fl/fl^/Cebpα::Cre (Cebpα-DC-Runx3^Δ^) or Runx3^fl/fl^/CD11c:Cre (CD11c-DC-Runx3^Δ^) mice, respectively. C57BL/6 Ly5.2 mice were purchased from Harlan Laboratories (Rehovot). C57BL/6 Ly5.1 mice and TCR-transgenic mice harboring ovalbumin (OVA)-specific CD4^+^ or CD8^+^ T cells were bred in the Weizmann animal facility. For generation of BM chimeras C57BL/6 Ly5.1 mice were lethally irradiated (1050 Rad) and reconstituted by intravenous injection of 1:1 mixture of C57BL/6 Ly5.1 and CD11c-DC-Runx3^Δ^ BM cells. Mice were analyzed 6-10 weeks later. This study was carried out in strict accordance with the recommendations in the Guide for the Care and Use of Laboratory Animals of the National Institutes of Health. The protocol was approved by the Committee on the Ethics of Animal Experiments of the Weizmann Institute of Science (Permit Number: 01190113-2). All surgery was performed under sodium pentobarbital anesthesia, and all efforts were made to minimize suffering.

### Flow cytometric analysis

 For analysis of BM DC precursors femurs and tibias BM cells were suspended in ACK erythrocyte-lysis buffer (0.15M NH4Cl, 0.1M KHCO3, and 1mM EDTA in PBS), washed and then stained with CD135 (A2F10), CD117 (2B8), CD115 (AFS98), and lineage Ab cocktail: CD11b (M1/70), CD3 (145-2C11), CD4 (GK1.5), CD8a (53-6.7), Gr1 (RB6-8C5), Sca-1 (D7), B220 (RA3-6B2), Ter-119, CD11c (N418), and NK1.1 (PK136). Splenic DC were obtained from spleens treated for 30 min with collagenase D (1mg/ml C5138 Sigma) and DNase I (20ug/ml Roche). DC were characterized using ACK-lyzed cell suspensions as CD11c^hi^MHCII^+^ CD4/CD8α or CD11b/Esam/CD8α. pDC were characterized as CD11c/PDCA1/B220 and monocytes as CD11b/CD115/Ly6c. DC subsets were analyzed for maturation by staining with CD40 (3/23) and CD86 (GL-1). Blood monocytes were detected as CD115/CD11b and Ly6C^+^ or Ly6C^-^ cells. DC were analyzed for apoptosis by annexin V and propidium iodide staining according to the manufacturer instruction (eBiosciences). All Abs were purchased from BioLegend or eBiosciences if not indicated otherwise. FACSAria flow cytometer (BD Biosciences) was used for cell sorting and forward scatter height versus forward scatter width appearance was used to exclude dublets. Analysis using LSRII flow cytometer (BD Biosciences) was conducted with FACSDiva Version 6.2 software (BD Biosciences). FACS data were further analyzed using FlowJo Version 9.3.2 software (TreeStar).

### RNA isolation and microarray gene expression analysis

 Total RNA from sorted DC was extracted using the miRNeasy Mini Kit (QIAGEN) including DNase digest (QIAGEN). RNA purity was assessed with a BioAnalyzer 2100 (Agilent Technologies). Samples of 250ng RNA from each isolated DC population was labeled and hybridized to Affymetrix mouse exon ST 1.0 microarrays according to manufacturer instructions. Microarrays were scanned using GeneChip scanner 3000 7G. Each experiment was conducted in duplicates or triplicates using RNA from independent FACS isolated cells. Statistical analysis was performed using the Partek® Genomics Suite (Partek Inc., St. Louis, Missouri 63141) software. CEL files (containing raw expression measurements) were imported to Partek GS. The data was preprocessed and normalized using the RMA (Robust Multichip Average) algorithm [21] with GC correction. To identify differentially expressed genes, One-Way ANOVA was applied. Differential gene lists were created by filtering the genes based on: absolute fold change >= 1.5, p <=0.05 and signal above background in at least one microarray (log_2_ intensity >=6.5). Log_2_ gene intensities were used for scatter plots (Partek) and matrix plots containing: correlation, histogram and scatter plots (using R language). Gene expression clusters for 224 transcripts were generated using log_2_ ratios with the Expander SOM tool [22]. These transcripts were differentially expressed in our and in [8] experiments and were above background in both datasets. Microarray data is available at Gene Expression Omnibus (GEO) under accession numbers GSE48589 (Runx3 function in CD4^+^ splenic DC) and GSE48590 (The affect of specific ablation of Runx3 from Esam splenic DC).

### ChIP-seq data acquisition and analysis

 Two biological replicate ChIP-Seq experiments were conducted for detection of Runx3-bound genomic regions using in-house anti Runx3 Ab and 30x10^6^ positive CD11c MACS isolated (Miltenyi Biotec) DC. These cells were further purified by FACS to CD11c^hi^MHCII^+^CD4^+^ cDC subset. Isolated cells were fixed in 1% formaldehyde and sonicated to yield DNA fragments of ~300bp according to standard procedures previously summarized in [23]. For immunoprecipitation, 40ul of anti-Runx3 Ab were added to 15 mL of diluted, fragmented chromatin; whole cell extract fragmented chromatin served as control. DNA was purified using QIAquick spin columns (QIAGEN). For ChIP-seq analysis, Illumina sequencing of short reads (50 bp) was performed in one lane of Hiseq 2000 using v2 clustering and sequencing reagents. For data analysis 70 million Runx3 IP and 74 million whole genome extract sequences were aligned uniquely to the mouse genome (mm9) using bowtie [24]. Bound regions were detected using MACS2 [25]. Runx3 bound peaks and coverage data (bigWig files) were uploaded to the UCSC genome browser [26]. GREAT algorithm was applied to determine genes corresponding to Runx3-bound peaks and ontology enrichment analysis [27]. Cistrome CEAS [28] was used to calculate average profile read near TSS and to generate venn diagrams. De-novo discovery of TF binding sites was conducted using DREME [29]. Analysis of co-bound zDC - Runx3 peaks was done using bedtools [30]. Statistical evaluation of fold enrichment distributions was done using the R package. ChIP-seq data is available at Gene Expression Omnibus (GEO) under accession number GSE48588 (Genome-wide maps of Runx3 bound regions in CD4^+^ splenic DC).

### RT–qPCR and Western blotting

 Total RNA was reverse-transcribed using miScript reverse transcription kit (QIAGEN) according to manufacturer's instructions. Quantitation of cDNAs was performed by qPCR using Roche LC480 *LightCycler* with sequence-specific primers and miScript SYBR Green PCR kit (QIAGEN). Target transcript quantification was calculated relative to ACTB mRNA, which served as an internal control. Standard errors were calculated using REST [31]. For Western blot analysis FACS isolated DC sub-populations were collected, washed once in PBS, proteins extracted with RIPA buffer and analyzed by Western blotting with anti Runx3 Ab [17]. GAPDH was used as an internal loading control.

### T-cell proliferation and Phagocytosis assays

 CD4^+^ and CD8^+^ OVA-specific T cells were isolated from spleens and lymph nodes of OT-II and OT-I TCR-transgenic mice and CD4 or CD8 cells isolated by MACS (Miltenyi Biotec). Cells were labeled with CFSE (Invitrogen) and coinjected into the tail veins of recipient mice (2 X 10^6^ cells/mouse). Twenty-four hours later, 20ug of soluble OVA (Sigma-Aldrich) per mouse was injected. Analysis of T-cell proliferation in spleens of recipient mice was performed 96 hours following T-cell transfer. For phagocytosis assay 200µl of 2.66% solid Fluoresbrite Carboxylate were injected into the tail veins with FITC labeled 0.5µm latex microspheres (Polysciences, Warrington, PA). Analysis of splenic DC microspheres uptake was performed 5 and 18 hours following injection. 

### Statistical analysis

 Statistical significance was determined with unpaired, two tailed Student’s t test. To identify differentially expressed genes, One-Way ANOVA was applied. Spearman correlation between microarray intensities was calculated using the R package. zDC peaks were found to be overlapping Runx3 peaks more than expected by chance using the hyperbrowser [32]. Specifically the test applied preserved the zDC regions and the number of Runx3 peaks and randomized (Monto Carlo test) the positions of Runx3 peaks with a constrain to the same chromosome and to a similar distance from the TSS. Same p value resulted in the opposite test (i.e. randomizing the zDC peaks). Kolmogorov-Smirnov test was applied on the Runx3 peaks fold enrichment distributions using the R package (ks.test) (http://www.R-project .org/). 

## Results

### Expression of Runx3 during DC lineage development

 To determine the developmental stages of the mononuclear phagocyte lineage where Runx3 expression is acquired, we took advantage of mice harboring a GFP reporter gene in their Runx3 loci [17] (Figure 1A). Specifically, we analyzed BM resident myeloid precursors (CMP, MDP, CDP and pre-DC), as well as mature myeloid splenocytes, of compound transgenic Runx3 P1/P2-GFP (Runx3^P1-GFP/P2-GFP^) mice that report activities of the two known Runx3 promoters P1 and P2 [17]. Analysis of BM-resident DC-progenitor populations revealed substantial variation in the abundance of Runx3-expressing cells. Among the common myeloid progenitor (CMP) cells (Lin^-^CD135^+^CD117^+^CD115^-^) ~50% were positive for Runx3-P1 and P2-GFP (Figure 1B and Figure S1A). The proportion of Runx3-P1 and P2-GFP expressing cells declined in MDP (Lin^-^CD135^+^CD117^+^CD115^int^) and CDP (Lin^-^CD135^+^CD117^low^CD115^+^) populations to ~30% and ~15%, respectively (Figure 1B and Figure S1A). Runx3-P1 and P2-GFP expression rose again to 25% in the pre-DC (Lin^-^CD135^+^CD11c^+^MHC-II^-^) population (Figure 1B and Figure S1A).

Reporter gene expression was absent from blood monocytes and plasmacytoid DC (pDC) (Figure S1B). Among splenic CD11c^hi^MHCII^+^ cDC Runx3/GFP expression was readily detected in CD4^+^CD8^-^ DC and DN populations, but absent from CD8^+^CD4^-^ DC (Figure 1C). Further analysis indicated that Runx3 expression was confined to CD11c^+^CD11b^+^ cDC (Figure S1C) with Runx3/GFP highly expressed in both Esam^hi^ and Esam^low^ sub-populations (Figure S1D). Lewis et al. recently proposed that Esam^hi^ and Esam^low^ DC subsets might originate along different pathways with only Esam^hi^CD11c^hi^CD11b^+^ DC, but not Esam^low^CD11c^hi^CD11b^+^ DC involving a CDP intermediate [8].

Interestingly, a similar reporter gene expression pattern was detected in cells derived from either Runx3^P1-AFP/+^ or Runx3^P2-EGFP/+^ mice (Figure S1A and E), indicating that *Runx3* expression in BM DC-progenitor subsets and mature CD11c^+^CD11b^+^ DC was driven by both P1 and P2 promoters. In summary, Runx3 expression was readily detected in sub-populations of BM CMP, MDP and pre-DC as well as in splenic CD11b^+^ DC, whereas Runx3 expression was absent from CD8^+^ DC, monocytes and pDC populations. 

### Runx3 regulates development of splenic CD11b^+^ DC

 Because DC development is associated with alterations in Runx3 expression levels (Figure S1A), we addressed its role in DC lineage specification and homeostasis. Mice lacking Runx3 starting from the CMP or Pre-DC stage were generated by crossing Runx3^fl/fl^ mice [17] onto Cebpα-Cre mice [19] and CD11c-Cre mice [20], respectively. To confirm efficient Runx3 ablation in these mouse models we performed a Western blot analysis on FACS-isolated CD4^+^ DC of wild-type (WT) mice, Runx3^fl/fl^ Runx3^fl/fl^/Cebpα::Cre (Cebpα-DC-Runx3^Δ^) and Runx3^fl/fl^/CD11c::Cre (CD11c-DC-Runx3^Δ^) mice (Figure S2A). Corroborating the findings with Runx3^P1/P2-GFP^ mice, Runx3 expression was confined to WT and Runx3^fl/fl^ CD4^+^DC, but absent from CD8^+^DC. In addition, CD4^+^DC isolated from Cebpα-DC-Runx3^Δ^ or CD11c-DC-Runx3^Δ^ mice lacked Runx3 protein (Figure S2A).

**Figure 2:**
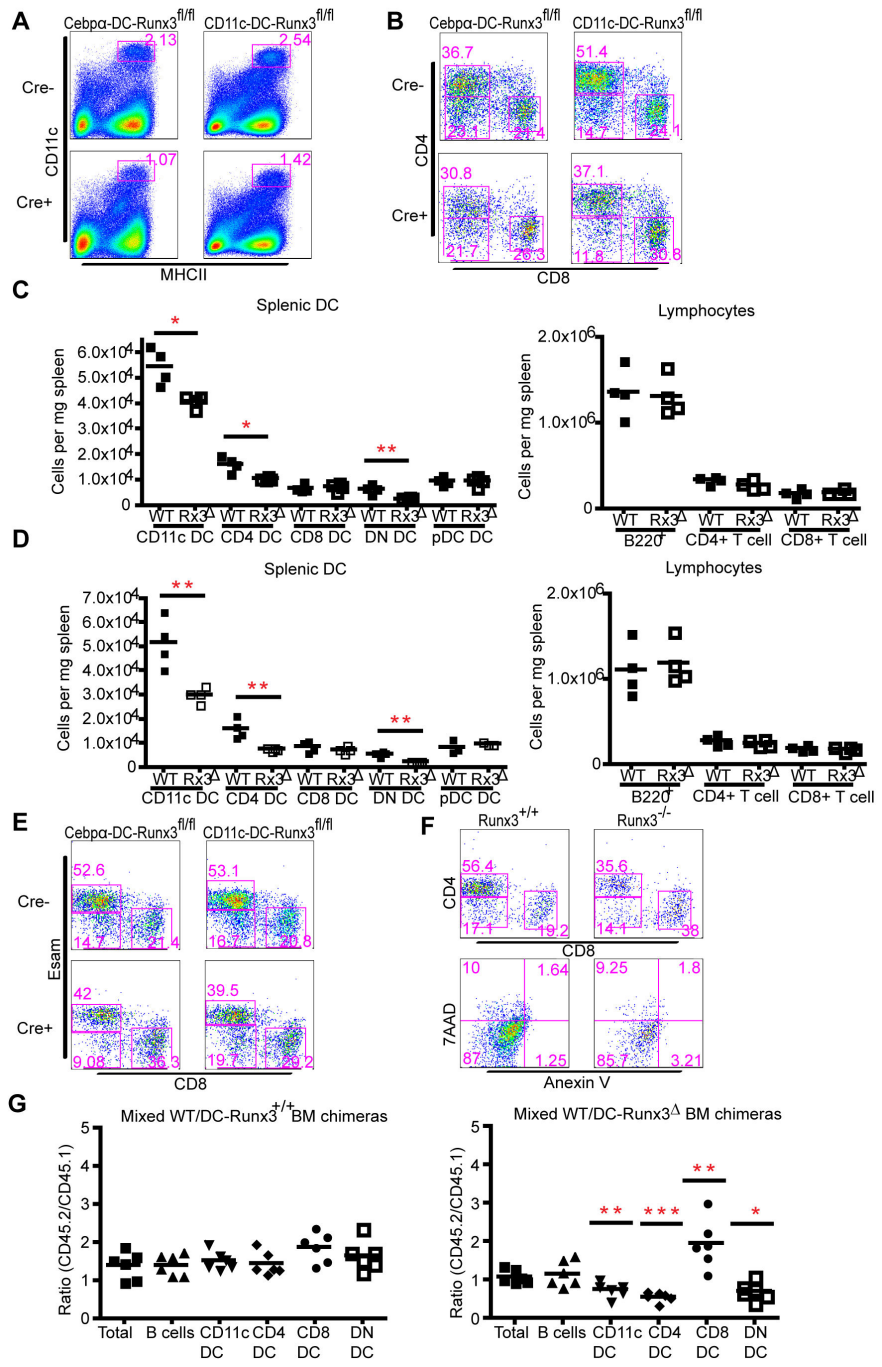
Runx3 is required for splenic CD11b^+^ DC lineage development. (A) Representative profiles of splenocytes isolated from Cebpα-DC-Runx3^Δ^ (left) or CD11c-DC-Runx3^Δ^ (right) mice stained for CD11c and MHCII. Comparisons between Cre- (upper) and Cre+ (lower) littermates are shown. (B) Representative profiles of gated CD11c^hi^MHCII^+^ splenic DC isolated from Cebpα-DC-Runx3^Δ^ (left) or CD11c-DC-Runx3^Δ^ (right), stained for CD4 and CD8. Comparisons between Cre- (upper) and Cre+ (lower) littermates are shown. Note the reduced proportion of CD4^+^ DC upon loss of Runx3. (C and D) Splenocytes were isolated from Cebpα-DC-Runx3^Δ^ (C), CD11c-DC-Runx3^Δ^ (D) or littermate control WT mice (n=4, 6-8 week old). Cells were counted and total cell numbers per mg tissue calculated and compared to that of littermate control. Each dot represents one independent animal. *P<0.05, **P<0.01, ***P<0.001 (Students two-tailed t test). Shown are results of one experiment out of two with the same finding. (E) Representative profiles of splenocytes isolated from Cebpα-DC-Runx3^Δ^ (left) or CD11c-DC-Runx3^Δ^ (right) mice gated for CD11c^hi^MHCII^+^ DC subset and analyzed for Esam and CD8. Comparisons between Cre^-^ (upper) and Cre^+^ (lower) littermates are shown. Shown representative of three independent experiments with one to two mice per group. (F) Lack of apoptosis among Runx3-deficient cDC. Representative profiles of Runx3^+/+^ (left panel) and Runx3^-/-^ (right panel) cDCs stained with Annexin-V and with 7AAD viability dye. Shown representative of two independent experiments with one to two mice per group. (G) Graphic summary of competitive BM-repopulation assay. Flow cytometric analysis of [Runx3^+/+^ (CD45.2) /WT (CD45.1) > WT (CD45.1)] (left) and [CD11c-DC-Runx3^Δ^ (CD45.2) /WT (CD45.1) > WT (CD45.1)] (right) chimeric mice 6-8 weeks after transplantation. CD45.2/CD45.1 ratios were calculated for each cell population. Values <1 indicate out-competition of the mutant by WT (CD45.1) cells, whereas values >1 show an advantage of Runx3^Δ^ (CD45.2) cells. Representative results from one out of two independent experiments are shown (mean ± SD) with 3 or more animals in each group. *P<0.05, **P<0.01, ***P<0.001 (Students two-tailed t test).

FACS analysis of Cebpα-DC-Runx3^Δ^ or CD11c-DC-Runx3^Δ^ splenocytes revealed a pronounced reduction in CD11c^hi^MHCII^+^ cells (Figure 2A). Detailed analysis of the splenic DC compartment indicated that loss of Runx3 specifically affected the CD11b^+^ subclass in both DC-Runx3^Δ^ mouse strains (Figure 2B-D). No other hematopoietic cell populations besides CD11c^hi^MHCII^+^CD11b^+^ DC seemed to be compromised, as evidenced by unaffected frequencies of B cells, T cells and pDC in both DC-Runx3^Δ^ mice compared to littermate controls (Figure 2C and D). Of note, the reduction in splenic CD4^+^ and DN DC affected both Esam^hi^ and Esam^low^ DC in the Cebpα-DC-Runx3^Δ^ and CD11c-DC-Runx3^Δ^ mouse strains (Figure 2E). The reduction of Esam^hi^ and Esam^low^ DC in these mice was however not associated with increased apoptosis (Figure 2F). FACS analysis of Runx3^-/-^ mice [18] that lacked Runx3 throughout myelopoiesis recapitulated the DC phenotype observed in the DC-Runx3^Δ^ mouse strains (Figure S2B-D). 

The finding that the two DC-lineage stage-specific Runx3^Δ^ mouse strains, as well as germ-line Runx3-mutants displayed a similar impairment of their DC compartment suggested that Runx3 may not be required at a specific lineage decision stage, but rather be involved in DC homeostasis. To address this issue we examined the ability of CD11c-DC-Runx3^Δ^ BM cells to replenish the DC compartment by conducting a competitive BM-repopulation assay. A 1:1 mixture of CD11c-DC-Runx3^Δ^ BM cells (CD45.2) and WT BM cells (CD45.1) was transferred into lethally irradiated recipient mice (CD45.1). Six to eight weeks following the transfer spleens from experimental (CD11c-DC-Runx3^Δ^/wt > wt) and control (Runx3^+/+^/wt > wt) chimeras were analyzed for the distribution of CD4^+^/CD8^+^ and DN DC. CD11c-DC-Runx3^+/+^ BM cells of the control group efficiently repopulated the three DC subsets successfully competing with the WT BM (Figure 2G left). In striking contrast, CD11c-DC-Runx3^Δ^ BM cells were severely impaired in generating CD11c^hi^MHCII^+^ cells (Figure 2G right). Specifically, CD4^+^Runx3^Δ^ DC and DN Runx3^Δ^ DC were found outcompeted by their WT counterparts, whereas Runx3^Δ^ CD8^+^ were overrepresented relative to WT CD8^+^ DC (Figure 2G right). Collectively, these findings demonstrate a cell-autonomous Runx3 function in CD4^+^ DC homeostasis with Runx3 loss significantly reducing splenic CD4^+^ DC numbers (Figure 2).

### Runx3 is required for lineage identity and homeostasis of Esam^hi^ DC

**Figure 3:**
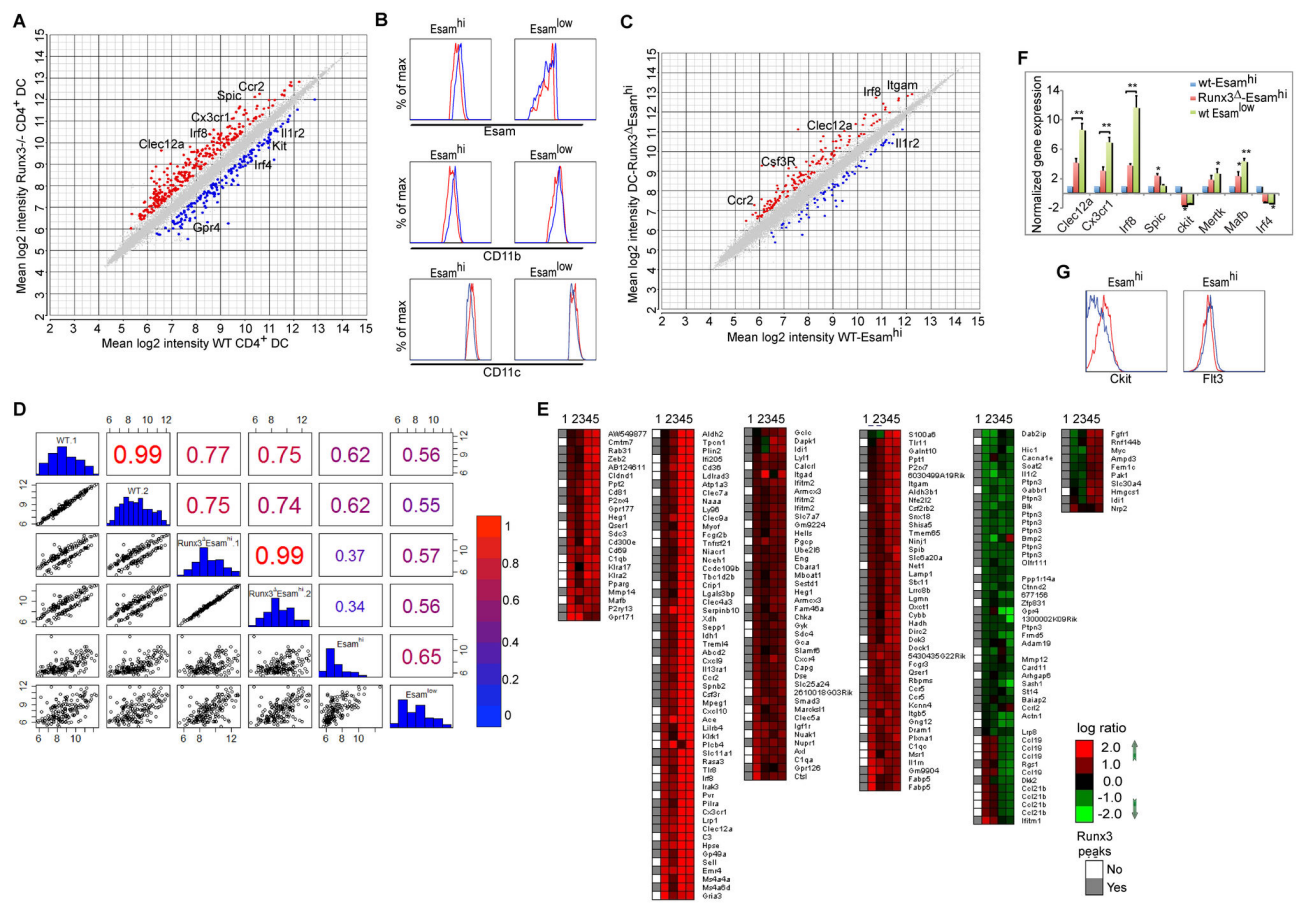
Alteration in CD4^+^ and Esam^hi^ DC gene expression due to loss of Runx3. (A) Scatter plot of differentially expressed genes in Runx3-deficient CD4^+^ DC and WT control DC. Splenic CD4^+^ DC from three Runx3^Δ^ and WT mice were isolated by FACS, RNA was isolated and subjected to microarray analysis. Shown are the mean intensities (log_2_) of WT and Runx3^Δ^ CD4^+^ DC. Genes that were up- or down- regulated due to Runx3^Δ^ are marked by red or blue, respectively. Differential expression cut-off was set to minimal absolute fold-change of 1.5, and p-value<=0.05. Selected relevant genes are indicated. (B) Expression of Esam (upper panel) CD11b (middle panel) and CD11c (lower panel) in splenic DC subsets from Runx3^+/+^ (Runx3^fl/fl^) mice (red line) and CD11c-DC-Runx3^Δ^ mice (blue line). (C) Scatter plot of differentially expressed genes in CD4^+^Esam^hi^ DC from WT and CD11c-DC-Runx3^Δ^ mice. Splenic Esam^hi^ DC were obtained from two individual mice. Genes that were up- or down- regulated due to Runx3^Δ^ are marked by red or blue, respectively. Differential expression cut-off was set to minimal absolute fold-change of 1.5, and p-value<=0.05. Selected relevant genes are indicated. (D) Gene expression log_2_ pairwise comparisons between WT-CD4^+^Esam^hi^, CD11c-Runx3^Δ^CD4^+^Esam^hi^ subsets and the published Esam experiment [8]. Compared are 162 genes differentially expressed in the DC-Runx3^Δ^Esam^hi^ experiment and in Lewis et al, 2011 Esam experiment [8]. The shown comparison of gene expression intensities indicates Spearman correlation values numerically and by color (above main diagonal), histograms of expression values (main diagonal) and scatter plots (below main diagonal). (E) Clusters showing Log_2_ ratio of gene expression of 224 genes (see Methods) correlated with Runx3 occupied genomic regions. Runx3 binding (up to a distance of 200kb from TSS) is presented as column No-1. Gene expression (Log_2_ ratio) was calculated for the following combination: column No-2 Runx3^-/-^CD4^+^ vs. WT-CD4^+^, column No-3 DC-Runx3^Δ^Esam^hi^ vs. CD4^+^, column No-4 WT Esam^low^ vs. Esam^hi^ and column No-5 DC-Rbpj^Δ^ vs. Esam^hi^. Levels of up or down regulation are color-coded. (F) RT-qPCR analysis of genes scored by differential gene expression of WT Esam^hi^ and Esam^low^ CD11b vs. Runx3^Δ^ Esam^hi^ populations. Data represents normalized expression values relative to WT Esam^hi^ sample (±SD of three assays from two biological repeats). (G) Histograms for Kit and Flt3 expression in Esam^hi^ DC subset. Red and blue lines represents WT CD11c (Runx3^fl/fl^) and CD11c-Runx3^Δ^ mice, respectively.

 To elucidate the cell-intrinsic regulatory role of Runx3 during splenic CD4^+^ DC homeostasis we sought to identify Runx3-responsive genes driving this process. Comparison of Runx3^-/-^ and WT CD4^+^ DC expression profiles revealed 412 differentially expressed genes (Figure 3A and Table S1). Significantly, several of these Runx3-responsive genes had previously been implicated in DC lineage development and homeostasis. For example, *Irf8* [10] and Spic/*PU*.*1* [33] were upregulated while *Irf4* [12] was downregulated in Runx3-deficient CD4^+^ DC (Figure 3A and Figure S3A). Moreover, comparative analysis of WT and Runx3^Δ^ CD4^+^DC revealed elevated levels of Esam and CD11b and reduced expression of CD11c in CD11c^+^-Runx3^Δ^ DC (Figure 3B and Figure S3B), suggesting that loss of Runx3 particularly affected the Esam^hi^ DC population. This conclusion is supported by the earlier findings that Runx3 is highly expressed in both CD11b^+^Esam^hi^ and CD11b^+^Esam^low^ DC (Figure S1C and D), but its loss preferentially affected the CD11b^+^/Esam^hi^ population (Figure 2). 

Subsequently, we analyzed differential gene expression in Runx3^Δ^ and WT CD11b^+^Esam^hi^ DC (Figure 3C). Interestingly, 66 (30%) of the 202 Runx3-responsive genes upregulated in Runx3^Δ^ CD11b^+^Esam^hi^ DC were previously identified as Esam^low^ DC specific [8] (Table S2). Consequently, the gene expression signature of Runx3^Δ^CD11b^+^Esam^hi^ DC corresponded to that of WT Esam^low^ DC (Spearman correlation values of 0.56 and 0.57 for Runx3^Δ^Esam^hi^/WTEsam^low^) (Figure 3D), while its correlation value with WT CD11b^+^Esam^hi^ (Runx3^Δ^Esam^hi^/WT-Esam^hi^) was markedly diminished to 0.34 and 0.37 (Figure 3D). These findings indicated that loss of Runx3 caused a shift of the CD11b^+^Esam^hi^ population towards a CD11b^+^Esam^low^ cell-identity manifested in altered gene expression. This conclusion was supported by the observation that the Runx3^-/-^CD4^+^ DC gene expression profile highly correlated with CD11b^+^Esam^low^ DC (Figure S3C), but also with that of Rbpj^Δ^CD11b^+^Esam^low^ DC [8]. These latter Esam^low^ DC were derived from mice lacking the Notch2-pathway TF Rbpj that regulates Esam expression [8]. Specifically, the correlation values of Runx3^-/-^Esam^hi^ to WT Esam^low^ DC and that of Runx3^-/-^Esam^hi^ to Rbpj^Δ^Esam^low^ DC were 0.72 and 0.70, respectively, compared to a correlation value of 0.52 for Runx3^-/-^Esam^hi^ to WT Esam^hi^ DC (Figure S3C). 

We next integrated the Runx3 ChIP-seq data (see below) with the differential gene expression results of Runx3^Δ^CD11b^+^Esam^hi^ DC and Runx3^-/-^CD4^+^ DC. Using this dataset we performed clustering analysis using 224 Runx3-responsive gene transcripts compared to WT CD11b^+^Esam^low^ and Rbpj^Δ^CD11b^+^Esam^low^ DC datasets [8]. Six clusters were identified (Figure 3E). Cluster 1-5 contained 186 Runx3-responsive genes (either down or up -regulated), of which 127 (~70%) were bound by Runx3. Importantly, clusters 1-4 included 156 genes that were upregulated in both Runx3-deficient CD11b^+^Esam^hi^ DC and CD4^+^ DC experiments and displayed a similar gene expression profile to that of WT CD11b^+^Esam^low^ DC previously reported [8]. This observation indicated that in WT CD11b^+^Esam^hi^ DC Runx3 negatively regulates these genes. We next used RT-qPCR analysis of RNA from Runx3^Δ^CD11b^+^Esam^hi^, WT CD11b^+^Esam^hi^ or WT CD11b^+^Esam^low^ DC or FACS analysis to validate the clustering microarray gene expression data. This analysis confirmed that key WT CD11b^+^Esam^low^ DC-specific genes, including *Clec12a, Cx*_*3*_ *cr1, Irf8* and *Flt3* were markedly upregulated in Runx3^Δ^CD11b^+^Esam^hi^ DC (Figure 3F and G) along with genes such as *Kit* and *Itgax* that were downregulated upon loss of Runx3 (Figure 3B and G). Taken together the data support the conclusion that Runx3 normally represses a number of critical genes to facilitate the maintenance of Esam^hi^ cell identity. Upon loss of Runx3, Runx3^Δ^Esam^hi^ DC acquired a gene expression profile characteristic to Esam^low^ DC. Interestingly, this cell identity shift of Esam^hi^ to Esam^low^ DC did not affect their maturation state, as evidenced by the level of activation markers (Figure S3D). 

### Identification of Runx3-regulated genes in splenic CD4^+^ DC

**Figure 4:**
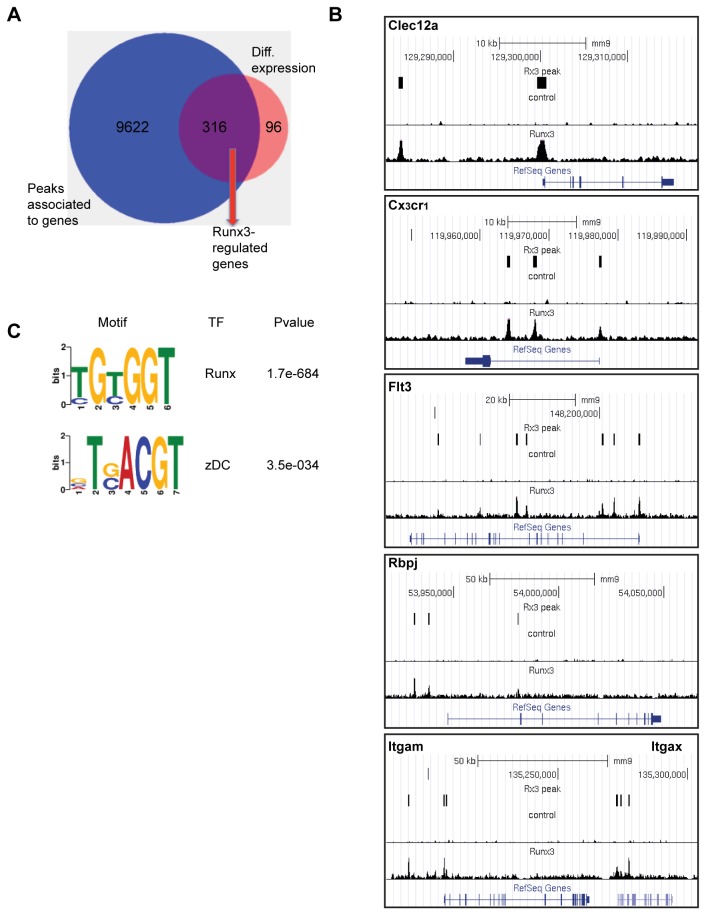
Analysis of Runx3 occupied genomic regions in splenic CD4^+^ DC. (A) Venn diagram summarizing the overlap between Runx3 ChIP-seq bound genes and differentially expressed genes in splenic CD4^+^ DC. (B) Runx3 binding-pattern on five genomic loci differentially expressed in CD4^+^ DC. Shown are ChIP-seq Runx3 peaks and the normalized read coverage of whole cell extract (top) and Runx3 ChIP (bottom) uploaded to UCSC Genome Browser mm9 genome assembly. The position of MACS2-identifird Runx3 peaks is indicated by rectangles. For each panel the scale bar and diagram of gene exon structure are presented on top or bottom, respectively. (C) Enriched RUNX and zDC TF motif among Runx3 bound regions. Motifs were identified *de-novo* using the MEME-ChIP software.

 We next sought to identify among Runx3-responsive genes those that were directly regulated by Runx3 and could therefore contribute to the cell-phenotypic shift of Esam^hi^ DC to Esam^low^ DC. ChIP-seq was conducted using FACS-isolated CD11c^hi^MHCII^+^CD4^+^ splenic WT DC and anti-Runx3 antibodies (Ab). Data analysis revealed that of the 15121 Runx3-bound regions 9938 were associated with annotated genes (Figure 4A). Location analysis of these Runx3-occupied regions relative to the nearest transcription start sites (TSS) of the annotated genes, revealed that ~50% were placed ±10kb from a gene TSS (Figure S4A and B). Runx3 ChIP-seq data analysis using GREAT [27] indicated that Runx3-bound regions were highly enriched for genes that belong to specific functional categories, including antigen processing and presentation (Figure S4C). To identify among the CD4^+^DC Runx3-responsive genes those that were Runx3-regulated we cross-analyzed Runx3 ChIP-Seq and differential gene expression datasets. Of the 412 Runx3-responsive genes 316 were bound by Runx3 and defined as Runx3-regulated (Figure 4A). Forty percent (127) of these Runx3-regulated genes belonged to the subset characteristic of WT CD11b^+^Esam^low^ DC in Runx3-deficient CD4^+^ DC (Figure 3E), underscoring the crucial role of Runx3 in specifying the Esam^hi^ DC gene expression signature. Among these Runx3-responsive genes, Runx3 was found to occupy the genomic loci of *Clec12a, Cx*_*3*_ *cr1, Flt3, Rbpj, Itgam* and *Itgax* (Figure 4B). 

Sequence analysis revealed that 86% of Runx3-occupied genomic regions contained the canonic RUNX motif TGTGGT (Figure 4C). Interestingly, Runx3-bound regions were also enriched for the motif TGACGT comprising the binding site of DC-specific TF zDC/zbtb46 [15] (Figure 4C). Moreover, comparison of the published zDC ChIP-seq dataset [15] to Runx3-occupied regions in splenic CD4^+^DC revealed that a high proportion (66%) of zDC bound genes was co-bound by Runx3 and that the zDC/Runx3 co-occupied regions were significantly enriched in Runx3 binding (Figure S4D). This observation is consistent with the possibility that Runx3 and zDC cooperate in regulating DC specific gene expression particularly as similar to Runx3, zDC participates in homeostasis of splenic CD4^+^DC [13].

### Runx3 is required for CD4^+^ DC-mediated T-cell priming

**Figure 5:**
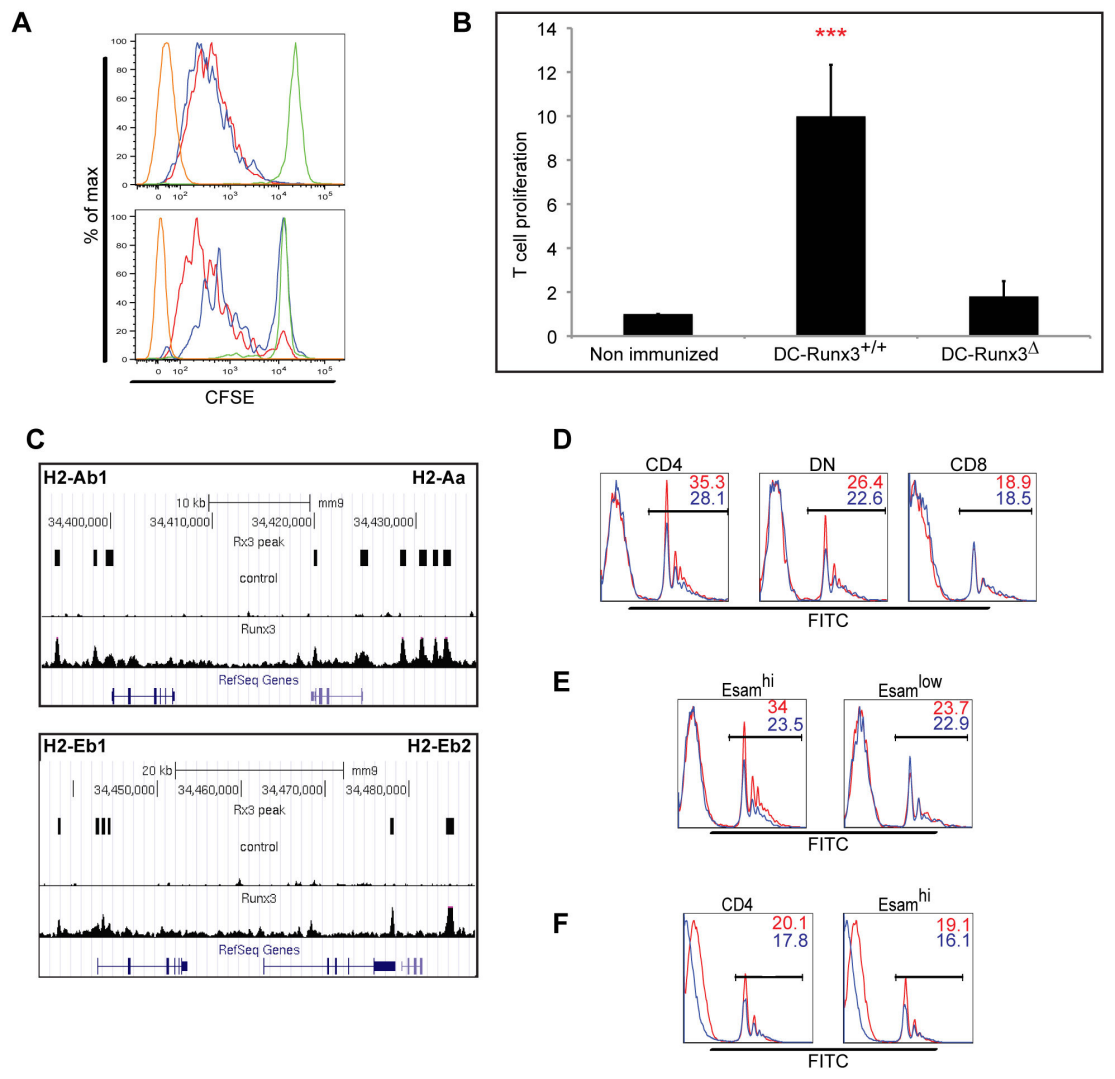
Loss of Runx3 abrogates functional activities of CD4^+^ DC. (A) CD4^+^ T cell priming in DC-Runx3^Δ^ mice *in* *vivo*. CFSE-labeled OVA-specific OT-I CD8^+^ T cells or OT-II CD4^+^ T cells were introduced to DC-Runx3^Δ^ or WT control littermate mice followed by immunization with OVA. Shown are flow cytometric analyses of transgenic T cell grafts recovered from immunized recipient mice. Proliferation of OT-I CD8^+^ T cells (top) and OT-II CD4^+^ T cells (bottom) cells in DC-Runx3^+/+^ (red) or DC-Runx3^Δ^ mice (blue) compared to PBS immunized mice (green) and non-labeled cells (brown). Shown representative of two independent experiments with two to three mice per group. (B) Total cell number of OT-II CD4+ T cell Proliferation quantification. PBS-immunized mice T cell proliferation is set as one (n=5). ***P < 0.001 (Student’s two-tailed t test). (C) Runx3 binding-pattern on MHCII genomic loci. Shown are ChIP-Seq tracing wiggle files uploaded to UCSC Genome Browser mm9 genome assembly and the normalized read coverage of whole cell extract (top) and Runx3 ChIP (bottom). The position of MACS2-identified Runx3 peaks is indicated by rectangles. For each panel the scale bar and diagram of gene exon structure are presented on top or bottom, respectively. (D and E) The capacity of splenic DC subtypes to phagocytose latex beads was analyzed in DC-Runx3Δ (blue) and WT (red) littermate mice 18h after i.v. injection of 1010 FITC-labeled 0.5μm latex beads. Shown are histograms of FITC-fluorescence in CD4+, CD8+ and DN DC subsets (D) or in Esamhi and Esamlow DC subsets (E). Shown representative of two independent experiments with two mice per group with the same finding. (F) The capacity of splenic DC to phagocytose latex beads was further analyzed using mixed BM chimera mice 18h after i.v. injection of 1010 FITC-labeled 0.5μm latex beads. Shown are histograms of FITC-fluorescence in CD4+ and Esamhi DC subsets gated on WT CD45.1 (red) or DC-Runx3Δ CD45.2 (red). Shown representative of two independent experiments with two mice per group.

 T cell priming and activation by DC link innate and adaptive immunity and modulate tolerance vs. immune response. CD4^+^ T cells are preferentially primed by CD4^+^DC in a MHC-II dependent manner [6,34]. CD4^+^ DC-dependent T cell priming was reported to be impaired in DC-Rbpj^Δ^ mice that lack CD11b^+^Esam^hi^ DC [8], raising the possibility that also the Runx3^Δ^-dependent phenotypic shift of CD4^+^Esam^hi^ to CD4^+^Esam^low^ DC would be associated with impaired T cell immunity. To address this scenario, we isolated Ovalbumin (OVA)-specific CD8^+^ and CD4^+^ T cells from TCR transgenic OT-I and OT-II mice, labeled them with CFSE, to allow monitoring of *in vivo* proliferation and transferred the cells into WT control and CD11c-DC-Runx3^Δ^ mice. Donor mice bore the congenic marker CD45.1, allowing detection of grafted cells in the CD45.2^+^ recipient mice. Four days following immunization with OVA the OT-I CD8^+^ T cell graft underwent pronounced proliferation in both WT and CD11c-DC-Runx3^Δ^ recipients (Figure 5A upper panel). Thus, the lack of Runx3 did not influence the cross-priming efficiency of CD8^+^ DC. In contrast, the response of grafted OT-II CD4^+^ T cells to the OVA challenge was impaired in CD11c-DC-Runx3^Δ^ mice as compared to control recipients (Figure 5A lower panel, B and Figure S5A), attesting to the important role of Runx3 in CD4^+^ DC ability to prime T cells. As Esam^low^CD11b^+^ DC lack MHC-II priming capacity [8] the diminished CD4^+^ T cell priming in DC-Runx3^Δ^ mice is most probably due to an intrinsic cell defect in the Esam^hi^CD4^+^ DC. Consistent with this conclusion, Runx3 bound (Figure 5C) and regulated expression (Figure S4C) of classical MHC-II genes and its loss could therefore affect MHC-II presentation. Together, the data indicate that Runx3 not only participates in promotion and maintenance of Esam^hi^ DC identity, but also contributes to their CD4^+^ T cell priming activity. 

### Reduced phagocytic capacity of Runx3-deficient splenic CD11b^+^ DC

 DC-mediated adaptive immune responses involve antigen phagocytosis followed by MHC-dependent T cell priming. The crucial contribution of Runx3 to the ability of Esam^hi^CD4^+^ DC to prime CD4^+^ T cells posed the question as to whether it was also required for phagocytic DC activity. To directly evaluate this possibility we injected 1x10^10^ FITC-labeled latex beads to WT and DC-Runx3^Δ^ mice, and monitored uptake of fluorescent beads by splenic DC, 5 and 18 hours later (Figure 5A and Figure S5B). Of note, Runx3^Δ^ CD4^+^/CD11b^+^ DC displayed a reduced capacity to phagocytose the beads compared to WT CD4^+^/CD11b^+^ DC, while Runx3^Δ^ CD8^+^ DC exhibited phagocytic capacity similar to WT DC (Figure 5D). Moreover, when we divided the CD11b^+^ DC population and analyzed the phagocytic ability of WT Esam^hi^ and Esam^low^ DC independently, we found that Esam^hi^ DC exhibited a higher phagocytosis potential than their Esam^low^ DC counterparts (Figure 5E and Figure 5B). However, this higher phagocytic capacity of WT Esam^hi^ DC diminished upon Runx3 ablation (Figure 5E and Figure S5C). Even more convincingly, injection of 1x10^10^ FITC-labeled latex beads into chimeras of a 1:1 mixture of CD11c-DC-Runx3^Δ^ BM cells (CD45.2) and WT BM cells (CD45.1) recapitulated the aberrant phagocytic phenotype of CD4^+^/CD11b^+^ DC (Figure 5F). Collectively these results indicated that in naive CD11b^+^/CD4^+^/Esam^hi^ DC, Runx3 regulates basic cDC functions including phagocytosis and antigen-processing. 

## Discussion

 Extensive efforts have been made over the past decade to elucidate the mechanisms underlying the production of DC-progenitors and to delineate the heterogeneity and functions of their downstream lineage cell subsets [7,35,36]. However, information about the role of specific TF in DC development, subset specification and homeostasis is still incomplete. Using cell-specific targeting of Runx3 at various stages of DC lineage development we show a cell-autonomous Runx3 requirement for splenic CD11b^+^Esam^hi^ DC homeostasis and function. In contrast to its critical role in CD4^+^Esam^hi^ DC specification and homeostasis, Runx3 contributions to early DC developmental stages are more modest. Accordingly, Runx3 expression levels are quite low among the DC precursor populations. Interestingly however, within the CMP, MDP and CDP progenitors Runx3 expression marks a small but distinct cell fraction comprising ~20-40% of the population. Whether the fraction of Runx3-expressing CMP or MDP are already committed to differentiate into the CD4^+^Esam^hi^ DC subset or rather have an equal potential to develop into CD8^+^ and CD11b^+^ DC is an important issue to address in the future. Nevertheless, this observation indicates that myeloid DC precursor populations may not be homogenous [7]. In mature splenic DC Runx3 expression is restricted to the CD11b^+^Esam^hi^ and CD11b^+^Esam^low^ subsets, whereas CD8^+^ DC are completely devoid of Runx3 expression. 

In good correlation with the expression pattern, inactivation of Runx3 at distinct stages of DC lineage development led to profound reduction of the CD4^+^Esam^hi^ DC subset. Interestingly, this Runx3-ablation-dependent reduction in CD4^+^Esam^hi^ DC was associated with extensive changes in CD4^+^Esam^hi^ DC gene expression, causing a shift in cell identity. Specifically, a considerable number of Runx3-regulated genes that are normally repressed in WT CD4^+^Esam^hi^ DC became activated in Runx3^Δ^CD4^+^Esam^hi^ DC. Consequently, Runx3^Δ^CD4^+^Esam^hi^ DC acquired a gene expression signature resembling that of WT Esam^low^ DC. Importantly, this gene expression shift of Runx3^Δ^CD4^+^Esam^hi^ led to acquisition of phenotypic features characterizing the WT Esam^low^ DC subset [8], including a compromised CD4^+^ T cell priming capacity, as well as decreased phagocytosis. 

The finding that in CD4^+^DC the zDC/Runx3 co-bound regions comprised more than 50% of the genomic regions occupied by this recently identified DC-specific TF [15] suggest that the two TF cooperate in driving the CD4^+^Esam^hi^ transcriptional program and may collaborate in repressing Runx3-regulated Esam^low^ DC genes. Of particular relevance to these recent findings is the observation that inactivation of the E2-2 TF during pDC maturation led to a major change in pDC gene expression [37,38] so that E2-2-deficient pDC displayed induced or repressed expression of genes characteristic to CD8^+^DC and pDC respectively [37]. Both TGF-β and Notch signaling pathways are important for DC development and function [20,39]. These two pathways crosstalk through protein-protein interaction of Notch intracellular domain with Smad3 [40]. Fainaru et al. have shown involvement of Runx3 in TGF-β dependent DC function [16] and Runx3 participates in Notch-mediated endothelial/mesenchymal transition [41]. Expression of the Esam receptor by CD11b^+^ DC is regulated by the Notch2-Rbpj signaling [8]. In this context, it is of note that Runx3^Δ^CD4^+^Esam^hi^ mice display elevated expression of Esam indicating Runx3 involvement in DC Notch2 signaling pathway. The idea that Runx3 participates in Notch2/TGF-β crosstalk during DC development is further supported by data documenting significant increased of Smad3 expression in Runx3^Δ^CD4^+^ DC. In summary, Runx3 play a crucial role in specifying Esam^hi^ DC identity and function. Accordingly, loss of Runx3 induces transcriptional reprogramming manifested in phenotypic shift of Esam^hi^ to Esam^low^ DC that compromises CD4^+^ T cell priming activity. 

## Supporting Information

Figure S1 **Analysis of Runx3 expression in splenic CD4^+^/CD11b^+^ DC and DC precursor CMP and MDP.**
(DOC)

Figure S2 **Data showing that Runx3 regulates splenic CD11b^+^ DC development.**
(DOC)

Figure S3 **Results demonstrating that loss of Runx3 alters CD4^+^ and Esam^hi^ DC gene expression.**
(DOC)

Figure S4 **Showing sequence analysis of Runx3 bound regions in splenic CD4^+^ DC.**
(DOC)

Figure S5 **Document the diminished capacity of splenic DC subtypes to phagocytose latex beads.**
(DOC)

Table S1 List of genes showing differential expression in Runx3-/- vs Runx3+/+ CD4+ DC measured by expression arrays, related to Figure 3A. Listed are genes that showed fold-change of at least 1.5 and p-value< 0.05.
(XLSX)

Table S2 List of genes showing differential expression in Esamhi-DC-Runx3Δ vs Esamhi-DC-Runx3+/+ measured by expression arrays, related to Figure 3c. Listed are genes that showed fold-change of at least 1.5 and p-value< 0.05.
(XLSX)
