# Supplementary material for: Transcriptional Reprogramming of CD11b+Esamhi Dendritic Cell Identity and Function by Loss of Runx3
Source: PLoS One. 2013 Oct 15;8(10):e77490. doi: 10.1371/journal.pone.0077490 (PMC3817345; doi:10.1371/journal.pone.0077490)
Supplement: Figure S1 — Analysis of Runx3 expression in splenic CD4+/CD11b+ DC and DC precursor CMP and MDP. (DOC) [file pone.0077490.s001.doc]

**SUPPORTING INFORMATION**

**Figure S1. Runx3 expression in splenic CD4^+^/CD11b^+^ DC and DC precursor CMP and MDP.** (**A**) Runx3 expression level and promoter-usage in BM DC-progenitor and mature splenic DC substes. Histograms summarizing average GFP expression resulting from either P1-AFP (blue) or P2-EGFP (red) in CMP, MDP, CDP, preDC BM DC progenitors and CD11c^+^MHCII^+^ cDC. (**B, C and D**) Flow cytometry of cells isolated from WT or Runx3^P1/P2-GFP^ mice. Histograms showing analysis of pDC and monocytes (B) or CD11c^+^CD11b^+^ DC (C) from Runx3^P1/P2-GFP^ mice (blue line) and Runx3^+/+^ littermate mice (red line). While both pDC and monocytes lack Runx3 expression and are negative for GFP the entire CD11c^+^CD11b^+^ DC population expresses Runx3. Results from one of two experiments with the same findings are shown. (D) Histograms showing GPF expression in Esam^hi^, Esam^low^ and CD8 DC subsets gated on CD11c^+^MHCII^+^ DC. Esam^hi^ and Esam^low^ DC express Runx3, wherease CD8^+^ DC do not. Results from one of two experiments with the same findings are shown. (**E**) Runx3 expression level and promoter-usage in mature splenic DC subsets. While splenic CD4^+^ and DN DC express Runx3-GFP from P1 and P2, CD8^+^ DC are GFP negative. Results from one of two experiments with the same findings are shown. Related to Figure 1.
