# Supplementary material for: Transcriptional Reprogramming of CD11b+Esamhi Dendritic Cell Identity and Function by Loss of Runx3
Source: PLoS One. 2013 Oct 15;8(10):e77490. doi: 10.1371/journal.pone.0077490 (PMC3817345; doi:10.1371/journal.pone.0077490)
Supplement: Figure S2 — Data showing that Runx3 regulates splenic CD11b+ DC development. (DOC) [file pone.0077490.s002.doc]

**SUPPORTING INFORMATION**

**Figure S2.** **Runx3 regulates splenic CD11b^+^ DC development.** (**A**) Western blot analysis using anti-Runx3 Abs. Protein cell extract from sorted CD4^+^ and CD8^+^ DC from Cebpα-DC-Runx3^Δ^ mice (upper panel) and CD11c-DC-Runx3^Δ^ (lower panel) mice. GAPDH was used as control of protein loadings. While high Runx3 levels are detected in WT and CD4^+^ DC the CD8^+^ DC subset and DC from Runx3^Δ^ mice are negative. (**B**) Shown are representative profiles of splenocytes isolated from WT and Runx3-/- mice stained for CD11c and MHCII (upper panel) and of gated CD11c^hi^MHCII^+^ DC stained for CD4 and CD8 (lower panel). Loss of Runx3 cause reduced percentage of CD4^+^ and DN DC. Results from one of two experiments with the same findings are shown. (**C and D**) Splenocytes isolated from WT and Runx3-/- mice (n=5 6-8 weeks old mice) were analyzed and subsets percentages and total cell numbers were calculated (cells/mg). Each dot represents an independent animal. *P<0.05, **P<0.01, ***P<0.001 (Students two-tailed t test). Results from one of two experiments with the same findings are shown. Related to Figure 2.
