# Supplementary material for: Transcriptional Reprogramming of CD11b+Esamhi Dendritic Cell Identity and Function by Loss of Runx3
Source: PLoS One. 2013 Oct 15;8(10):e77490. doi: 10.1371/journal.pone.0077490 (PMC3817345; doi:10.1371/journal.pone.0077490)
Supplement: Figure S3 — Results demonstrating that loss of Runx3 alters CD4+ and Esamhi DC gene expression. (DOC) [file pone.0077490.s003.doc]

**SUPPORTING INFORMATION**

**Figure S3. Loss of Runx3 alters CD4^+^ and Esam^hi^ DC gene expression.** (**A**) RT-qPCR analysis of genes scored by differential gene expression analysis of WT vs. Runx3^-/-^ CD4^+^ DC populations. Data represents normalized expression values relative to WT Esam^hi^ sample (±SD of three assays from two biological repeats). (**B**) Mean fluorescence intensity (MFI) of CD11c, CD11b and Esam expression in WT (CD45.1) and CD11c-DC-Runx3^Δ^ (CD45.2) Esam^hi^ DC subsets isolated from mixed BM chimeras. Each dot plot represents an independent animal. **P<0.01 and ***P<0.001 (Students two-tailed t test). Results from one of two experiments with the same findings are shown. (**C**) Gene expression (log_2)_ pairwise comparison (mean of 3 biological repeats) between WT CD4^+^ and Runx3^-/-^ CD4^+^ to WT Esam^hi^, Esam^low^ and DC-Rbpj^Δ^ subsets. Genes analyzed (335) were found to be differential expressed in the Runx3^Δ^ CD4^+^ experiment and above background in the published (Lewis et al, 2011) Esam gene expression experiment. The shown comparison of gene expression intensities indicates Spearman correlation values numerically and by color (above main diagonal), histograms of expression values (main diagonal) and scatter plots (below main diagonal). (**D**) Shown are histograms of CD40 and CD86 expression in Esam^hi^ DC subset from WT (Runx3^fl/fl^) and CD11c-DC-Runx3^Δ^ mice presented as red and blue lines, respectively. Related to Figure 3.
