# Supplementary material for: Transcriptional Reprogramming of CD11b+Esamhi Dendritic Cell Identity and Function by Loss of Runx3
Source: PLoS One. 2013 Oct 15;8(10):e77490. doi: 10.1371/journal.pone.0077490 (PMC3817345; doi:10.1371/journal.pone.0077490)
Supplement: Figure S4 — Showing sequence analysis of Runx3 bound regions in splenic CD4+ DC. (DOC) [file pone.0077490.s004.doc]

**SUPPORTING INFORMATION**

**Figure S4. Sequence analysis of Runx3 bound regions in splenic CD4^+^ DC.** (**A**) Frequency of bound Runx3 proximal to annotated TSS. Fifty % of Runx3 bound regions appear ±10kb of annotated TSS. (**B**) Runx3 bound regions at the TSS show an increase in average signal profile. (**C**) GREAT analysis of Runx3 ChIP-seq bound regions in CD4^+^DC revealed highly significant overrepresentation of genes with important roles in DC functions. (**D**) Co-binding of zDC and Runx3 in CD4^+^DC. Histograms summarizing the distribution of fold enrichment (Runx3 coverage versus whole cell extract coverage) within the Runx3 ChIP-seq peaks (red), relative to zDC/Runx3 co-bound regions (blue) indicating enrichment for Runx3 binding in co-bound peaks. Fold enrichment distributions were found to be significantly different using the Kolmogorov-Smirnov test (D = 0.1653, p-value < 2.2e-16). Related to Figure 4.
