# Supplementary material for: Transcriptional Reprogramming of CD11b+Esamhi Dendritic Cell Identity and Function by Loss of Runx3
Source: PLoS One. 2013 Oct 15;8(10):e77490. doi: 10.1371/journal.pone.0077490 (PMC3817345; doi:10.1371/journal.pone.0077490)
Supplement: Figure S5 — Document the diminished capacity of splenic DC subtypes to phagocytose latex beads. (DOC) [file pone.0077490.s005.doc]

**SUPPORTING INFORMATION**

**Figure S5. Functional deficiencies of Runx3^Δ^ CD4^+^ DC.** (**A**)

Quantification of CFSE mean fluorescence intensity (MFI) of proliferated OTII CD4^+^ T cells. Each dot represents an independent animal. **P<0.01 (Students two-tailed t test). The capacity of splenic DC subtypes to phagocytose latex beads was analyzed in DC-Runx3^Δ^ (blue) and WT (red) littermates mice 5h after iv injection of 10^10^ FITC-labeled 0.5μm latex beads. Shown are histograms of FITC-fluorescence in (**B**) CD4^+^, CD8^+^ and DN DC subsets (**C**) or in Esam^hi^ and Esam^low^ DC subsets. Results from one of two experiments with the same findings are shown. Related to Figure 5.
